# Supplementary material for: Petiveria alliacea Reduces Tumor Burden and Metastasis and Regulates the Peripheral Immune Response in a Murine Myeloid Leukemia Model
Source: Int J Mol Sci. 2023 Aug 19;24(16):12972. doi: 10.3390/ijms241612972 (PMC10454792; doi:10.3390/ijms241612972)
Supplement: Supplementary file 1 [file ijms-24-12972-s001.zip › ijms-2527679-supplementary.pdf]

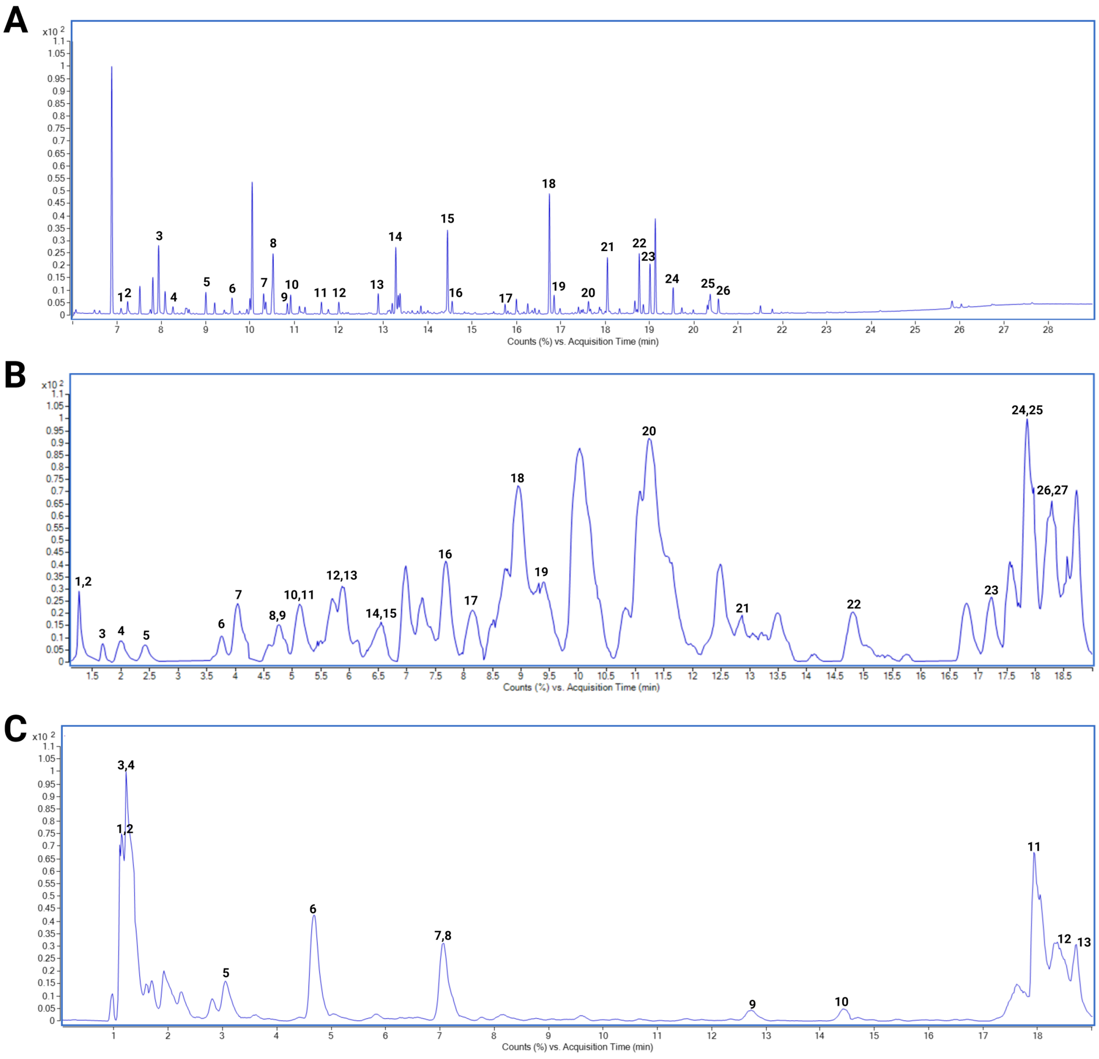

Figure S1. Chemical characterization of Esperanza extract. **A.** GC-MS total compound chromatogram. Peak identification: (1) Glycolic acid; (2) Valine; (3) Oxalic acid; (4) Benzyl alcohol; (5) Malonic acid; (6) Benzoic Acid; (7) Isoleucine; (8) Succinic Acid; (9) Glyceric acid; (10) Uracil; (11) Threonine; (12) Alanine; (13) Malic acid; (14) Aspartic acid; (15) Glutamic acid; (16) Phenylalanine; (17) Xylitol; (18) Citric acid (19) Myristic acid; (20) Tyramine; (21) Mannitol ; (22) Palmitic acid; (23) Glucaric acid ; (24) Myo-Inositol; (25) Linolenic acid; (26) L-Tryptophan. **B.** LC-MS (-) total compound chromatogram. Peak identification: (1) Glucaric acid; (2) Galactaric acid; (3) Isocitric acid; (4) Citric acid; (5) Xanthine; (6) Guanosine; (7) Gly-Leu-OH/Ala-Val-OH/Tyr Asp; (8) Vanillic acid; (9) Phenylalanine; (10) Pantothenate; (11) Glutamyltyrosine; (12) Toluenesulfonic acid; (13) Phospho ribosyl dimethylbenzimidazole; (14) Ferulic acid; (15) Tryptophan; (16) Pantetheine phosphate; (17) Fulgidic acid; (18) Sulfooxyjasmonate; (19) Acetylleucine;(20) Xanthotoxol glucoside; 21) Bikoeniquinone A; (23) O-Caffeoyl-O-methylquinic acid; (24) Linusitamarin; (24) Corchorifatty acid F; (25) Trihydroxy octadecenoic acid; (26) Oxylipin-3; (27) Ethyl-oxocyclopent-1enyl hydroxyundecenoic acid. **C.** LC-MS (+) total compound chromatogram. Peak identification: (1) Methyl (hydroxymethyl)pyrrolidine-carboxylate; (2) Germacradiene-acetoxy-diol; (3) Amino-methylenehexanoic acid; (4) Proline betaine; (5) Phenylacetaldehyde; (6) Methylbenzaldehyde; (7) Indoleacrylic acid; (8) Indole-carboxaldehyde; (9) Phenylvaleric acid; (10) Cyanolipid 16:0 ester; (11) Funtumine; (12) Oxo-octadecatetraenoic acid; (13) Auxin b.

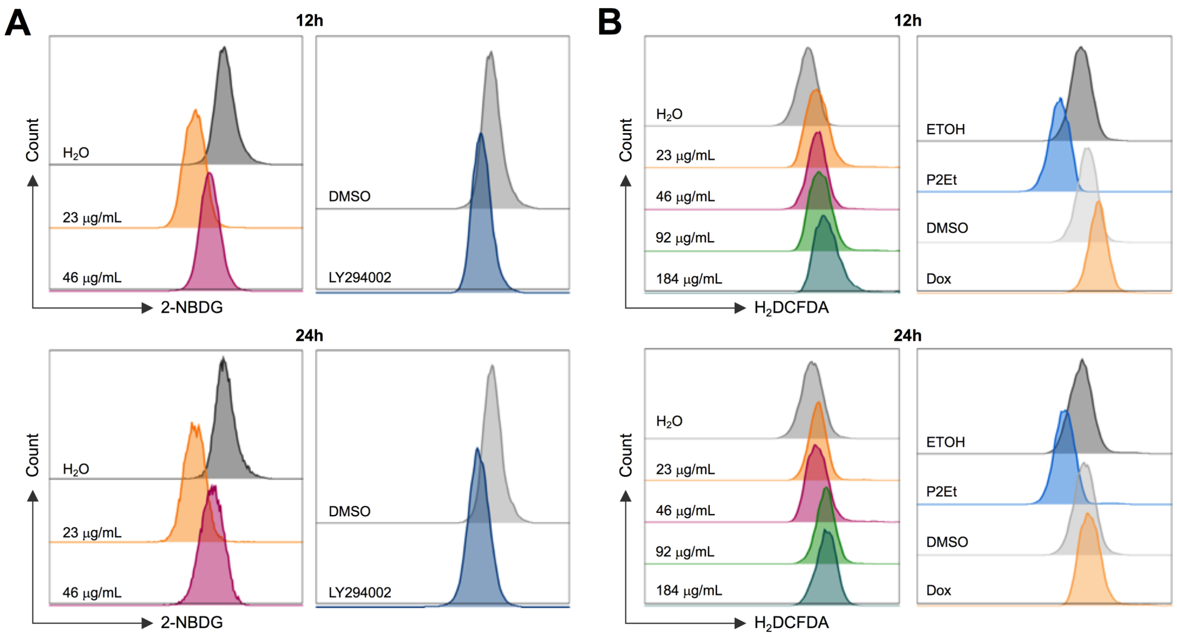

Figure S2. Representative flow cytometry histograms for live, DA-3/ER-GM cells incubated with 2-NBDG **(A)** or H_2DCFDA **(B)** after treatments with different concentrations of Esperanza extract and controls. H_2O (Esperanza vehicle), DMSO (LY294002 or Doxorubicin vehicle), ETOH (P2Et vehicle) LY294002 (control of decreased glucose uptake), Doxorubicin (pro-oxidant control), P2Et (anti-oxidant control).

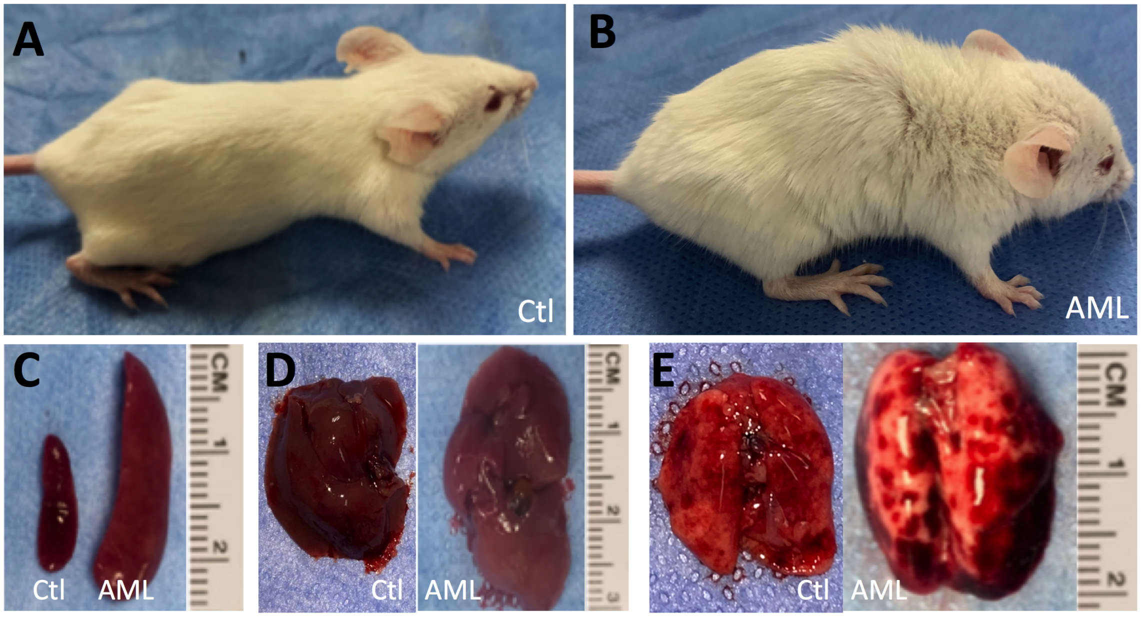

Figure S3. Physical and macroscopic observations in the necropsy of animals inoculated with 5 x 10^4 DA-3/ER-GM cells. Representative appearance of **(A)** healthy control (Ctl) and **(B)** mouse injected with 5 x 10^4 DA-3/ER-GM (AML) on the day of euthanasia (Day 12). **C.** Representative image of spleens weights from Ctl and AML mice at 12 days from DA-3/ER-GM cells injection. **D.** Representative image of livers weights from Ctl and AML mice at 12 days from DA-3/ER-GM cells injection. **E.** Representative image of lungs weights from Ctl and AML mice at 12 days from DA-3/ER-GM cells injection.

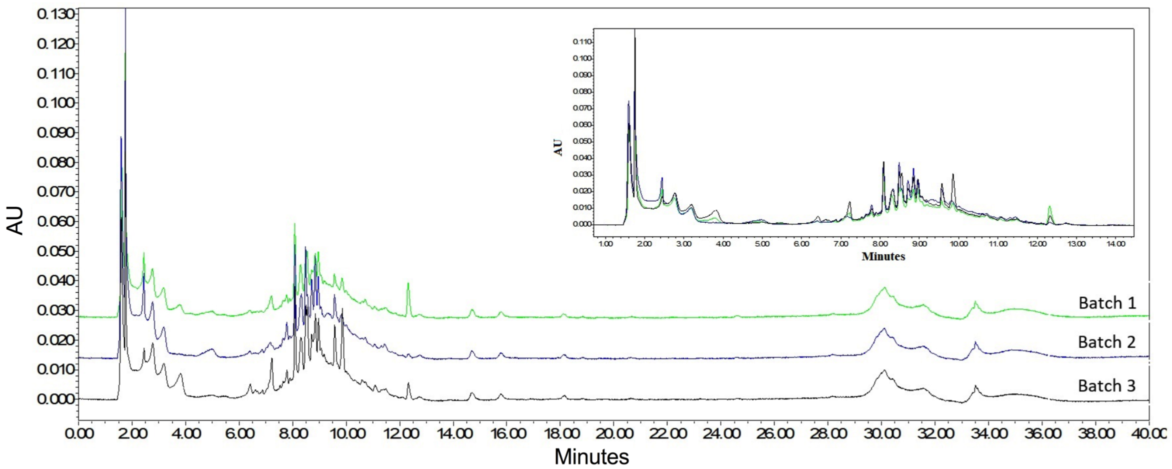

Figure S4. Chromatographic analysis of the different batches of the Esperanza extract. UPLC-PDA chromatogram at 274 nm of three batches of Esperanza aqueous extract manufactured under GMP conditions.

Table S1. Chemical composition of the aqueous extract of *P. alliacea* analyzed by GC-MS

Compound name	Molecular formula	Molecular weight	Retention time (min)	Analytical platform	ID Level	Relative abundance (%)**
Carboxylic acids and derivatives						
Citric acid	$C_6H_8O_7$	192.0270	16.75*	GC-MS	2	12.97
Glutamic acid	$C_5H_9NO_4$	147.0532	14.45*	GC-MS	2	10.82
Aspartic acid	$C_4H_7NO_4$	133.0375	13.28*	GC-MS	2	7.09
Oxalic acid	$C_2H_2O_4$	89.9953	7.94	GC-MS	2	6.47
Succinic Acid	$C_4H_6O_4$	118.0266	10.52	GC-MS	2	6
Alanine	$C_3H_7NO_2$	89.0477	12.08*	GC-MS	2	3.01
Malonic acid	$C_3H_4O_4$	104.0110	9.00*	GC-MS	2	2.5
Valine	$C_5H_{11}NO_2$	117.0790	7.24*	GC-MS	2	2.38
Threonine	$C_4H_9NO_3$	119.0582	11.61*	GC-MS	2	2.28
Leucine	$C_6H_{13}NO_2$	131.0946	10.00*	GC-MS	2	2.1
Glycine	$C_2H_5NO_2$	75.0320	10.49*	GC-MS	2	1.8
Proline	$C_5H_9NO_2$	115.0633	10.35*	GC-MS	2	1.79
Aminobutyric acid	$C_4H_9NO_2$	103.0633	13.38	GC-MS	2	1.75
Isoleucine	$C_6H_{13}NO_2$	131.0946	10.31*	GC-MS	2	1.69
Oxoproline	$C_5H_7NO_3$	129.0426	13.29*	GC-MS	2	1.19
Phenylalanine	$C_9H_{11}NO_2$	165.0790	14.55*	GC-MS	2	1.13
Serine	$C_3H_7NO_3$	105.0426	11.24*	GC-MS	2	0.9
Aminolevulinic acid	$C_5H_9NO_3$	131.0582	9.93*	GC-MS	2	0.43
Tyrosine	$C_9H_{11}NO_3$	181.0739	17.33	GC-MS	2	0.42
Tartronic acid	$C_3H_4O_5$	120.0059	11.17*	GC-MS	2	0.15
Pentanedioic acid	$C_5H_8O_4$	132.0423	13.93*	GC-MS	2	0.07
Propanoic acid	$C_3H_6O_2$	74.0368	17.62*	GC-MS	2	0.05
Homoserine	$C_4H_9NO_3$	119.0582	12.40*	GC-MS	2	0.05
Fumaric Acid	$C_4H_4O_4$	116.0110	10.95	GC-MS	2	0.03
Hygric acid	$C_6H_{11}NO_2$	129.0790	8.44	GC-MS	2	0.02
Aminobutanoic acid	$C_4H_9NO_2$	103.0633	6.36*	GC-MS	2	0.01

Aconitic acid	$C_6H_6O_6$	174.0164	15.87*	GC-MS	2	0.11
Mimosine	$C_8H_{10}N_2O_4$	198.0641	8.85	GC-MS	2	0.04
Fatty Acyls						
Palmitic acid	$C_{16}H_{32}O_2$	256.2402	18.77*	GC-MS	2	6.14
Linolenic acid	$C_{18}H_{30}O_2$	278.2246	20.37*	GC-MS	2	1.71
Hydroxy-methylbutyric acid	$C_5H_{10}O_3$	118.0630	8.45	GC-MS	2	0.13
Butanoic acid	$C_4H_8O_2$	88.0524	12.16*	GC-MS	2	0.12
Heptadecanoic Acid	$C_{17}H_{34}O_2$	270.2559	19.68*	GC-MS	2	0.09
Dodecanoic acid	$C_{12}H_{24}O_2$	200.1776	14.71*	GC-MS	2	0.09
Caproic acid	$C_6H_{12}O_2$	116.0837	7.00	GC-MS	2	0.09
Azelaic acid	$C_9H_{16}O_4$	188.1049	16.33*	GC-MS	2	0.07
Arachidic acid	$C_{20}H_{40}O_2$	312.3028	22.20	GC-MS	2	0.05
Palmitoleic acid	$C_{16}H_{30}O_2$	254.2246	18.59*	GC-MS	2	0.05
Myristic acid	$C_{14}H_{28}O_2$	228.2089	16.83*	GC-MS	2	0.05
Citramalic acid	$C_5H_8O_5$	148.0372	12.72*	GC-MS	2	0.05
Hydroxyisovaleric acid	$C_5H_{10}O_3$	118.0630	9.08	GC-MS	2	0.03
Docosanoic Acid	$C_{22}H_{44}O_2$	340.3341	23.74*	GC-MS	2	0.01
Octadecenoic acid	$C_{18}H_{34}O_2$	282.2559	20.35	GC-MS	2	0.9
Octadecadienoic acid	$C_{18}H_{32}O_2$	280.2402	20.30*	GC-MS	2	0.25
Organooxygen compounds						
Myo-Inositol	$C_6H_{12}O_6$	180.0634	19.54*	GC-MS	2	2.1
Erythritol	$C_4H_{10}O_4$	122.0579	13.20	GC-MS	2	1.08
Fructose	$C_6H_{12}NO_6$	194.0665	17.40*	GC-MS	2	0.84
Allose	$C_6H_{12}O_6$	180.0634	19.81*	GC-MS	2	0.77
Xylitol	$C_5H_{12}O_5$	152.0685	15.75*	GC-MS	2	0.76
Acetaldehyde	C_3H_7NO	73.0528	17.91*	GC-MS	2	0.26
Mannitol	$C_6H_{14}O_6$	182.0790	18.01*	GC-MS	2	0.19
Galactose	$C_6H_{12}O_6$	180.0634	17.68*	GC-MS	2	0.11
Melezitose	$C_{18}H_{32}O_{16}$	504.1690	24.20*	GC-MS	2	0.1
Pantothenic acid	$C_9H_{17}NO_5$	219.1107	18.46*	GC-MS	2	0.05
Ribose	$C_5H_{10}O_5$	150.0528	15.26*	GC-MS	2	0.03

Hydroxybenzaldehyde	$C_7H_6O_2$	122.0368	12.87	GC-MS	2	0.01
Hydroxy acids and derivatives						
Malic acid	$C_4H_6O_5$	134.0215	12.89*	GC-MS	2	1.78
Hydroxybutyric acid	$C_4H_8O_3$	104.0473	8.36*	GC-MS	2	0.15
Benzene and substituted derivatives						
Benzoic Acid	$C_7H_6O_2$	122.0368	9.59*	GC-MS	2	1.69
Tyramine	$C_8H_{11}NO$	137.0841	17.63*	GC-MS	2	1.15
Benzyl alcohol	C_7H_8O	108.0575	8.26*	GC-MS	2	0.18
Phenylethanol	$C_8H_{10}O$	122.0732	9.47	GC-MS	2	0.1
Gallic acid	$C_7H_6O_5$	170.0215	18.10*	GC-MS	2	0.03
Hydroxybenzoic acid	$C_7H_6O_3$	138.0317	14.49*	GC-MS	2	0.08
Diazines						
Uracil	$C_4H_4N_2O_2$	112.0273	10.91*	GC-MS	2	1.6
Pyrimidine	$C_4H_4N_2$	80.0374	11.76	GC-MS	2	0.37
Imidazopyrimidines						
Hypoxanthine	$C_5H_4N_4O$	136.0385	16.51	GC-MS	2	0.32
Xanthine	$C_5H_4N_4O_2$	152.0334	18.72*	GC-MS	2	0.19
Adenine	$C_5H_5N_5$	135.0545	17.16*	GC-MS	2	0.03
Phenylpropanoic acids						
Phenyllactic acid	$C_9H_{10}O_3$	166.0630	14.06	GC-MS	2	0.15
Prenol lipids						
Glucuronide	$C_{26}H_{36}O_8$	476.2410	21.98*	GC-MS	2	0.09
Phytol	$C_{20}H_{40}O$	296.3079	19.99*	GC-MS	2	0.33
Keto acids and derivatives						
Pyruvic Acid	$C_3H_4O_3$	88.0160	6.70*	GC-MS	2	0.03
Organonitrogen compounds						
Ethanolamine	C_2H_7NO	61.0528	6.39*	GC-MS	2	0.03
Saturated hydrocarbons						
Tridecane	$C_{13}H_{28}$	184.2191	19.25	GC-MS	2	0.01
Cinnamic acids and derivatives						
Hydroxycinnamic acid	$C_9H_8O_3$	164.0473	17.83	GC-MS	2	0.01

Cinnamic acid	$C_9H_8O_2$	148.0524	13.50*	GC-MS	2	0.02
Quinic acid	$C_7H_{12}O_6$	192.0634	17.26	GC-MS	2	0.08
Pinitol	$C_7H_{14}O_6$	194.079	16.99*	GC-MS	2	0.52
Glyceric acid	$C_3H_6O_4$	106.0266	10.84*	GC-MS	2	0.85
Phenols						
Vanillylmandelic acid	$C_9H_{10}O_5$	198.0528	16.84	GC-MS	2	0.01
Tyrosol	$C_8H_{10}O_2$	138.0681	13.84	GC-MS	2	0.02
Flavonoids						
Cyanidin	$C_{15}H_{11}O_6$	287.0556	16.66	GC-MS	2	0.01
Pyrroles						
Pyrrole-carboxylic acid	$C_5H_5NO_2$	111.032	11.14*	GC-MS	2	0.01
Hydroxy acids and derivatives						
Glycolic acid	$C_2H_4O_3$	76.016	7.09*	GC-MS	2	0.55

GC-MS: Gas chromatography coupled to mass spectrometry. *The retention time corresponds to the sililated compound. **Relative abundance calculated with the chromatographic areas found within the same analytical platform.

Table S2. Chemical composition of the aqueous extract of *P. alliacea* analyzed by LC-MS

Compound name	Molecular formula	Molecular weight	Retention time (min)	Error (ppm)	Adduct	Ionization	Analytical platform	ID Level	Relative abundance (%)*
Carboxylic acids and derivatives									
Amino-methylenehexanoic acid	$C_7H_{13}NO_2$	143.0946	1.24	5	$[M+H]^+$	ESI+	LC-MS	4	6.44
Proline betaine	$C_7H_{13}NO_2$	143.0946	1.24	5	$[M+H]^+$	ESI+	LC-MS	4	6.44
Azetidinecarboxylic acid	$C_4H_7NO_2$	101.0477	1.69	1	$[M+H]^+$	ESI+	LC-MS	2	0.54
Deoxy-fructosylphenylalanine	$C_{15}H_{21}NO_7$	327.1318	4.8	2	$[M+H]^+$	ESI+	LC-MS	2	0.47
Deoxymugineic acid	$C_{12}H_{20}N_2O_7$	304.1271	1.56	3	$[M+H]^+$	ESI+	LC-MS	3	0.4
Phenylalanine	$C_9H_{11}NO_2$	165.079	4.67	3	$[M+H]^+$	ESI+	LC-MS	2	0.22
Pyroglutamic acid	$C_5H_7NO_3$	129.0426	1.52	4	$[M+H]^+$	ESI+	LC-MS	2	0.11
Glutamylphenylalanine	$C_{14}H_{18}N_2O_5$	294.1216	8.12	2	$[M+H]^+$	ESI+	LC-MS	2	0.1
Glutamyltyrosine	$C_{14}H_{18}N_2O_6$	310.1165	5.33	3	$[M+H]^+$	ESI+	LC-MS	2	0.05
Pantetheine phosphate	$C_{11}H_{23}N_2O_7PS$	358.0964	7.7	8	$[M+HCOOH-H]^-$	ESI-	LC-MS	3	1.25
Dipeptide	$C_{13}H_{16}N_2O_6$	296.1008	4.04	1	[M+Cl]-	ESI-	LC-MS	3	0.87
Citric acid	$C_6H_8O_7$	192.027	1.96	3	$[M-H]^-$	ESI-	LC-MS	2	0.33
Isocitric acid	$C_6H_8O_7$	192.027	1.35	4	$[M-H]^-$	ESI-	LC-MS	2	0.08
Tyrosine	$C_9H_{11}NO_3$	181.0739	3.06	3	$[M-H]^-$	ESI-	LC-MS	2	0.06
Phenylalanine	$C_9H_{11}NO_2$	165.079	4.61	2	$[M-H]^-$	ESI-	LC-MS	2	0.13
Acetylleucine	$C_8H1_5NO_3$	173.1052	9.48	2	$[M-H]^-$	ESI-	LC-MS	2	0.13
Glutamyltyrosine	$C_{14}H_{18}N_2O_6$	310.1165	5.32	1	$[M-H]^-$	ESI-	LC-MS	2	0.03
Tryptophan	$C_{11}H_{12}N_2O_2$	204.0899	6.97	0	$[M-H]^-$	ESI-	LC-MS/GC-MS	2	0.23
Fatty Acyls									
Oxo-octadecatetraenoic acid	$C_{18}H_{26}O_3$	290.1882	18.35	3	$[M+H]^+$	ESI+	LC-MS	3	1.42
Colnelenic acid	$C_{18}H_{28}O_3$	292.2038	18.17	1	$[M+H]^+$	ESI+	LC-MS	2	0.99
Octadecatrien-ynoic acid	$C_{18}H_{26}O_2$	274.1933	18.18	3	$[M+H]^+$	ESI+	LC-MS	2	0.75
Cyanolipid 16:0 ester	$C_{21}H_{37}NO_2$	335.2824	14.42	3	$[M+H]^+$	ESI+	LC-MS	3	0.72
Corchorifatty acid F	$C_{18}H_{32}O_5$	328.2250	18.15	4	$[M+Na]^+$	ESI+	LC-MS	3	0.55

Phenylvaleric acid	$C_{11}H_{14}O_2$	178.0994	12.69	3	$[M+H]^+$	ESI+	LC-MS	2	0.51
Corchorifatty acid F	$C_{18}H_{32}O_5$	328.225	17.84	2	$[M-H]^-$	ESI-	LC-MS	2	1.64
Fulgidic acid	$C_{18}H_{32}O_5$	328.225	8.15	2	$[M-H]^-$	ESI-	LC-MS	2	1.11
Oxylipin-3	$C_{18}H_{34}O_5$	330.2406	18.31	0	$[M-H]^-$	ESI-	LC-MS	3	0.99
Ethyl-oxocyclopentenyl hydroxyundecenoic acid	$C_{18}H_{28}O_4$	308.1988	18.35	3	$[M-H]^-$	ESI-	LC-MS	2	0.77
Trihydroxy octadecenoic acid	$C_{18}H_{34}O_5$	330.2406	17.99	1	$[M-H]^-$	ESI-	LC-MS	2	0.64
Sulfoxyjasmonate	$C_{12}H_{18}O_7S$	306.0773	8.98	1	$[M-H]^-$	ESI-	LC-MS	2	0.06
Azelaic acid	$C_9H_{16}O_4$	188.1049	14.23	2	$[M-H]^-$	ESI-	LC-MS	2	0.08
Organooxygen compounds									
Linusitamarin	$C_{17}H_{22}O_9$	370.1264	17.22	10	$[M-H]^-$	ESI-	LC-MS	4	0.92
Methoxy hydroxyphenylglycol glucuronide	$C_{15}H_{20}O_{10}$	360.1056	6.55	9	$[M-H]^-$	ESI-	LC-MS	4	0.52
Glucaric acid	$C_6H_{10}O_8$	210.0376	1.13	0	$[M-H]^-$	ESI-	LC-MS/GC-MS	2	0.1/4.5
Galactaric acid	$C_6H_{10}O_8$	210.0376	1.13	3	$[M-H]^-$	ESI-	LC-MS	2	0.1
Pantothenate	$C_9H_{17}NO_5$	219.1107	5.26	1	$[M-H]^-$	ESI-	LC-MS	2	0.06
Benzene and substituted derivatives									
Methylbenzaldehyde	C_8H_8O	120.0575	4.69	3	$[M+H-H_2O]^+$	ESI+	LC-MS	2	2.58
Dimethylaniline-N-oxide	$C_8H_{11}NO$	137.0841	5.05	4	$[M+H-H_2O]^+$	ESI+	LC-MS	4	0.52
Salicylic acid	$C_8H_9NO_2$	151.0633	2.24*	3	$[M+H]^+$	ESI+	LC-MS/GC-MS	2	0.43
Phenylacetaldehyde	C_8H_8O	120.0575	3.05	5	$[M+H]^+$	ESI+	LC-MS	2	2.81
Hydroxybenzaldehyde	$C_7H_6O_2$	122.0368	2.82	4	$[M+H]^+$	ESI+	LC-MS	2	0.31
Toluenesulfonic acid	$C_7H_8O_3S$	172.0194	5.69	3	$[M-H]^-$	ESI-	LC-MS	2	0.72
Vanillic acid	$C_8H_8O_4$	168.0423	4.57*	3	$[M-H]^-$	ESI-	LC-MS/GC-MS	2	0.15
Hydroxybenzoic acid	$C_7H_6O_3$	138.0317	14.31	4	$[M-H]^-$	ESI-	LC-MS	2	0.17
Imidazopyrimidines									
Guanine	$C_5H_5N_5O$	151.0494	3.87	3	$[M+H]^+$	ESI+	LC-MS	2	0.15
Erythro-eritadenine	$C_9H_{11}N_5O_4$	253.0811	1.74	1	$[M+H]^+$	ESI+	LC-MS	2	0.04
Carbamoyl-threonyladenosine	$C_{15}H_{20}N_6O_8$	412.1343	7.77	3	$[M+H]^+$	ESI+	LC-MS	2	0.03
Xanthine	$C_5H_4N_4O_2$	152.0334	2.26	4	$[M-H]^-$	ESI-	LC-MS	2	0.18
Indoles and derivatives									
Indoleacrylic acid	$C_{11}H_9NO_2$	187.0633	7.04	3	$[M+H]^+$	ESI+	LC-MS	3	1.93

Indole-carboxaldehyde	C_9H_7NO	145.0528	7.04	5	$[M+H]^+$	ESI+	LC-MS	2	1.2
Indole	C_8H_7N	117.0578	7.06	6	$[M+H]^+$	ESI+	LC-MS	2	0.95
Bikoeniquinone A	$C_{27}H_{20}N_2O_3$	420.1474	12.87	4	$[M+HCOOH-H]^-$	ESI-	LC-MS	3	0.55
Phenylpropanoic acids									
Pyridines and derivatives									
Nicotinate	$C_6H_5NO_2$	123.032	1.67	4	$[M+H]^+$	ESI+	LC-MS/GC-MS	2	0.32
Isonicotinic acid	$C_6H_5NO_2$	123.032	2.11	4	$[M+H]^+$	ESI+	LC-MS	2	0.2
Prenol lipids									
Germacradiene-acetoxy-diol	$C_{17}H_{28}O_4$	296.1988	1.16	2	$[M+Na]^+$	ESI+	LC-MS	3	2.04
Auxin b	$C_{18}H_{30}O_4$	310.2144	18.8	4	$[M+Na]^+$	ESI+	LC-MS	3	1.01
Cuminyl alcohol	$C_{10}H_{14}O$	150.1045	12.73	3	$[M+H-H_2O]^+$	ESI+	LC-MS	2	0.16
Sterebin A	$C_{18}H_{30}O_4$	310.2144	18.25	8	$[M-H]^-$	ESI-	LC-MS	2	1.15
Cinnamic acids and derivatives									
Ferulic acid	$C_{10}H_{10}O_4$	194.0579	6.72	2	$[M-H]^-$/ $M^{+\cdot}$	ESI-/EI	LC-MS	2	0.07
O-Feruloylquinic acid/O-Caffeoyl-O-methylquinic acid	$C_{17}H_{20}O_9$	368.1107	14.8	10	$[M-H]^-$	ESI-	LC-MS	3	0.89
Organooxygen compounds									
Pantothenate	$C_9H_{17}NO_5$	219.1107	5.28	3	$[M+H]^+$	ESI+	LC-MS	2	0.049
Phenols									
Phenol	C_6H_6O	94.0419	4.68	7	$[M+H]^+$	ESI+	LC-MS	3	0.72
Moupinamide	$C_{18}H_{19}NO_4$	313.1314	16.15	2	$[M+H]^+$/[M-H]-	ESI+/-	LC-MS	2	0.1
Flavonoids									
Hydroxy-tetramethoxyflavone	C19H18O7	358.1053	12.39	0	$[M-H]^-$	ESI-	LC-MS	3	0.034
Pyrroles									
Methyl (hydroxymethyl)pyrrolidine-carboxylate	$C_7H_{13}NO_3$	159.0895	1.14	4	$[M+K]^+$	ESI+	LC-MS	2	2.33
Steroids									
Funtumine	$C_{21}H_{35}NO$	317.2719	18.11	2	$[M+H]^+$	ESI+	LC-MS	3	1.44
Quinolines and derivatives									
Methylquinoline	$C_{10}H_9N$	143.0735	7.04	4	$[M+H]^+$	ESI+	LC-MS	2	0.84
Non-metal oxoanionic compounds									
Sulfate	H_2SO_4	97.9674	5.14	4	$[M-H]^-$	ESI-	LC-MS	3	2.11

Nucleosides. nucleotides. and analogues									
Phosphoribosyl dimethylbenzimidazole	$C_{14}H_{19}N_2O_7P$	358.093	5.9	1	$[M-H]^-$	ESI-	LC-MS	3	1.46
Guanosine	$C_{10}H_{13}N_5O_5$	283.0917	3.66	1	$[M-H]^-$	ESI-	LC-MS	2	0.06
Coumarins and derivatives									
Xanthotoxol glucoside	$C_{17}H_{16}O_9$	364.0794	11.09	9	$[M-H]^-$	ESI-	LC-MS	3	0.75

ESI: Electrospray Ionization; LC-MS: Liquid chromatography coupled to mass spectrometry. *Relative abundance calculated with the chromatographic areas found within the same analytical platform.
